# Re-evaluation of chronic hepatitis B and hepatitis C patients lost to follow-up: results of the Northern Holland hepatitis retrieval project

**DOI:** 10.1186/s41124-018-0032-9

**Published:** 2018-01-31

**Authors:** N. Beekmans, M. Klemt-Kropp

**Affiliations:** Department of Gastroenterology and Hepatology, Northwest Clinics, Alkmaar, The Netherlands

**Keywords:** Hepatitis B, Hepatitis C, Viral hepatitis elimination, Public health

## Abstract

**Background:**

Many persons infected with Hepatitis B virus (HBV) and Hepatitis C virus (HCV) in the past are now lost to follow-up. The aim of the Northern Holland Hepatitis Retrieval Project (NHHRP) is to retrieve and re-evaluate persons previously diagnosed with HBV or HCV and bring them back into care. Chronic HBV infection was defined as two positive Hepatitis B surface antigen (HBsAg) tests within 6 months and chronic HCV infection with 2 positive HCV RNA tests by polymerase chain reaction (PCR).

**Methods:**

Data files of the local public health services and microbiology laboratory were explored to identify all registered HBV and HCV cases in the Alkmaar region, the Netherlands, for the past 15 years. Identified cases were compared with patients currently known in our hospital. Patients without follow-up in primary or hospital care were approached via their primary health care physician and invited for evaluation at our hospital

**Results:**

In total, 552 cases of HBV were identified. 356 (64.5%) had no follow-up. Only 113/356 (31.7%) were eligible for retrieval and 44.2% were evaluated in our hospital resulting in a change of management in 22/50 (44%) of patients. Four hundred ninety nine cases of HCV were identified, 150/499 (30.1%) were lost to follow-up. Only 20/150 (13.3%) were eligible for retrieval and 4/20 (20%) were evaluated at our clinic. Resulting in a change of management in 3/4 (75%).

**Conclusion:**

Only a limited part of HBV and HCV persons lost to follow-up is eligible for retrieval, nonetheless re-evaluation of these persons will lead to a change of management in the majority of persons.

## Background

Infection with hepatitis B virus (HBV) and hepatitis C virus (HCV) is present worldwide and a leading cause of liver-related morbidity and mortality. It is estimated that 257 million persons worldwide are living with chronic HBV infection and 71 million persons with chronic HCV infection [1]. The World Health Organization (WHO) estimates that viral hepatitis was responsible for 1.34 million deaths in 2015, 96% of these deaths were due to long-term complications of untreated HBV and HCV infection [1].

In high-endemic HBV areas, such as sub-Saharan Africa and East-Asia, perinatal transmission is the most common mode of infection resulting in chronic HBV in more than 90% of infected neonates. In low-endemic countries such as Western Europe and North-America the majority of acute HBV infections occur during adolescence or adulthood and predominantly via sexual activity or intravenous drug use. In immunocompetent adults, less than 1% of acute HBV infections will progress to chronic HBV infection [2]. Acute HCV infection however, will lead to chronic infection in 55-80% of patients [3]. The predominant forms of transmission of HCV are unsafe therapeutic injections and blood transfusion in developing countries. In developed countries, injection drug use and unsafe sexual activities of HIV positive are the main forms of transmission [4].

In order to prevent long-term complications, it is important to achieve an adequate viral suppression or even elimination. Successful antiviral therapy can delay progression to cirrhosis and hepatocellular carcinoma and improve survival [2, 5, 6]. In chronic HBV infection, antiviral therapy is indicated if levels of HBV DNA are elevated, elevated alanine aminotransferase (ALT) values and/or at least moderate liver necroinflammation or fibrosis [7]. .The main goals of antiviral treatment are long-term suppression of HBV replication and hepatic inflammation and thereby preventing progression to cirrhosis and hepatocellular carcinoma. Treatment with antiviral therapy in HBV is often lifelong. In patients without an direct indication for treatment it is required to check ALT levels and viral load every 6-12 months in order to monitor and prevent progression to cirrhosis and hepatocellular carcinoma [7].

The primary goal of treatment in HCV infection is to cure HCV infection. The endpoint of therapy is a sustained viral response defined as undetectable HCV RNA 12 weeks after treatment completion [8]. Every patient with HCV infection has an indication for antiviral therapy according to the Dutch Hepatitis C guideline [9]. If cirrhosis already is present, even after curation, regular follow-up is necessary to screen for hepatocellular carcinoma.

The WHO set the ambitious goal to eliminate HBV and HCV as public health threat by 2030.

To accomplish this goal it is important to detect all persons infected with HBV or HCV. Several screening strategies are deployed in high-risk and low-risk population to detect infected persons. However, many persons previously diagnosed with HBV or HCV are lost to follow-up in primary and/or hospital care. This is an important target group. After all, the chance of spontaneous clearance is low and therefore an indication for antiviral treatment or strict follow-up might exist.

The goal of the Northern Holland Hepatitis Retrieval Project (NHHRP) is to focus on persons previously diagnosed with chronic HBV or HCV who are now lost to follow-up. We aimed to retrieve these persons and bring them back into care. To the best of our knowledge this is the first structured retrieval project within a geographic region. Furthermore, we aim to create a frame-work for other local or even nationwide retrieval programmes.

## Methods

Data files of the local public health services and the local microbiology laboratory were explored to identify all registered cases of chronic HBV and HCV in our region for the past 15 years. The Alkmaar region in Northern Holland covers about 300.000 inhabitants.

A full-time fellow was appointed to retrieve all data and evaluate patients at our clinic from January 2016 to September 2016.

Persons with a chronic HBV infection were defined as two positive Hepatitis B surface antigen (HBsAg) tests within 6 months. Chronic HCV infection was defined as two positive anti-HCV tests within 6 months confirmed with a positive HCV RNA test. Identified cases were compared with patients currently under follow-up in hospital or primary care. Persons without follow-up appointment were considered lost to follow up.

Due to Dutch privacy regulations, it was not allowed to directly contact identified persons since no active medical treatment agreement existed. Therefore, we informed the registered primary health care physician of identified persons and requested to refer them for evaluation at our clinic. Hence, only persons with updated contact details (registered primary health care physician, address) were eligible for retrieval. If persons moved to another area and no longer were registered at a primary health care physician in our region it was not possible for us to contact them. We were not allowed to use other databases, for instance municipality databases, to search for updated contact details.

Persons with severe comorbidity and an estimated survival of less than 1 year were excluded. If there was no referral within 2 months, we send a reminder to the primary health care physician.

Re-evaluation at our hospital included physical examination, blood examination for Alanine-aminotransferase (ALT), complete serology, HBV DNA load and genotype determination, and transient elastography of the liver. A management advice was proposed based on the outcome of evaluation in accordance to the Dutch guidelines on treatment of HBV and HCV infection [10].

The goal of the NHHRP was to retrieve patients lost to follow-up. In order to evaluate the feasibility of such a retrieval project, we aimed to evaluate the following outcomes:

- The number of persons with chronic HBV or HCV infection lost to follow-up
- The number of persons lost to follow-up we were able to retrieve and responded to our invitation for evaluation
- The number of persons for whom re-evaluation resulted in a change of management

Results were evaluated using descriptive statistics.

The Northern Holland Hepatitis Retrieval Project was approved by the local ethics committee.

## Results

### Chronic HBV

Concerning patients with chronic HBV we identified 552 cases and in our region.

**Fig. 1:**
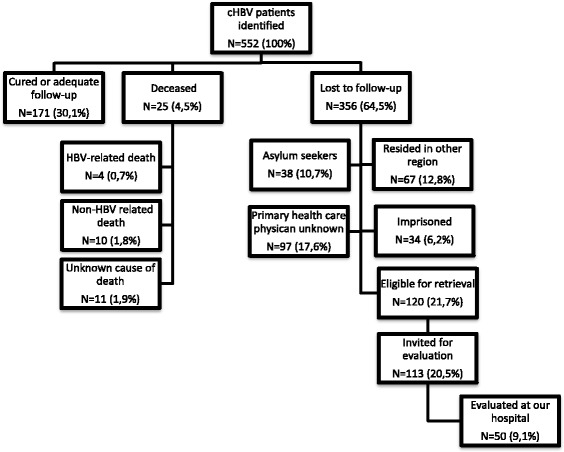
Retrieval of chronic HBV patients, The flow-chart reflects the results of retrieval of 552 chronic HBV patients

In total, 356/552 (64.5%) HBV patients had no follow-up in primary or hospital care (see Fig. 1). Only 120/356 (33.7%) were eligible for retrieval and after consulting their primary care physician, 113/120 (94.1%) were invited for evaluation. The remaining 7 patients suffered from severe comorbidity. The majority of patients with HBV were not eligible for retrieval because of various reasons. In 97/236 (41.1%) patients the primary health care physician was unknown, 34/236 (14,4%) were imprisoned, 38/236 (16,1%) were asylum seekers with unknown address and 67/236 (28.4%) now resided in another region.

In total, 50 of the 113 (44.2%) responded to the invitation and were evaluated at our hospital. Patient characteristics and outcome of evaluation are described in Table 1.

**Table 1:** HBV patient characteristics and outcome of evaluation

	*N* = 50 (100%)
Sex	
Male	22 (44%)
Female	28 (56%)
Age (years) (mean ± SD)	48 ± 9.7
Migrational background	
Dutch / WE	4 (8%)
EE/EA	12 (24%)
NA	2 (4%)
SSA	7 (14%)
SEA	23 (46%)
LA	2 (4%)
ALT (U/l) (mean + SD) combined with HBV DNA levels (IU/mL)	
Normal (0-34 U/l) and HBV DNA < 2.0 · 10^4^	44 (88%)
ALT >1xULN and HBV DNA < 2.0 · 10^4^	3 (6%)
ALT >1xULN and HBV DNA > 2,0 · 10^4^	3 (6%)
Fibrosis stage	
Unknown	2 (4%)
F0-F1	45 (90%)
F2-F3	3 (6%)
Management advice	
No Follow-up needed	3 (6%)
ALT levels every 6-12 months	25 (50%)
ALT levels and HCC screening every 6-12 months	14 (28%)
Strict follow-up every 3-6 months	5 (10%)
Antiviral therapy	3 (6%)

*WE* Western Europe, *EE* Eastern Europe, *EA* EuroAsia, *NA* North Africa, *SSA* Sub-Saharan Africa, *SEA* South East Asia, *LA* Latin America, *ALT* Alanine-aminotransferase, *ULN* Upper limit of normal, *HCC* Hepatocellular carcinoma

All patients were hepatitis B e antigen (HBeAg) negative. Evaluation resulted in a change of management in 22/50 (44%) of patients. An additional indication for HCC-screening was recommended in 14/50 (28%), 5/50 (10%) had an indication for strict follow-up and 3/50 (6%) an indication to start antiviral therapy. The remaining 25/50 (50%) were advised to have a 6-12 monthly check of ALT levels and in 3/50 (6%) patients viral load was undetectable.

### Chronic HCV

**Fig. 2:**
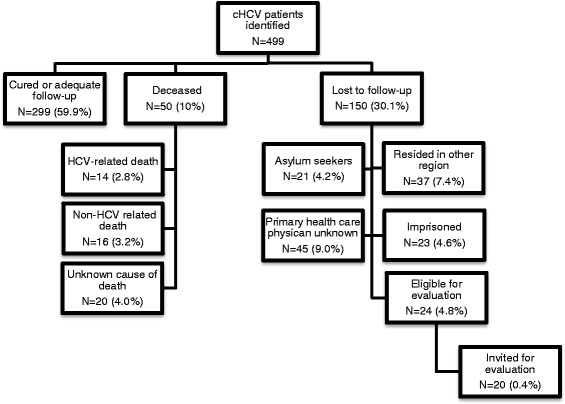
Retrieval of chronic HCV patients, The flow-charts reflect the results of retrieval of 499 chronic HCV patients

In total, 499 cases of chronic HCV were identified in our region (see Fig. 2).

In 150/499 (30.1%) persons, no follow-up was scheduled in primary or hospital care. Only 24/150 (16%) were eligible for retrieval and after consultation of their primary health care physician, 20/24 (83.3%) were invited for evaluation. Of the 126 persons not eligible for retrieval, in 45/126 (35.7%) their primary health care physician was unknown, 21/126 (16.7%) were asylum seekers with unknown address, 23/126 (18.3%) were imprisoned, 37/126 (29.4%) resided in another region.

Patient characteristics and outcome of evaluation are described in Table 2.

**Table 2:** HCV patient characteristics and outcome of evaluation

	*N* = 4 (100%)
Sex	
Male	2 (50%)
Female	2 (50%)
Age (years) (mean ± SD)	59 ± 21
Migrational background	
Dutch / WE	3 (75%)
EE/EA	1 (25%)
ALT (U/l) (mean + SD)	35 ± 18
Normal (0-34 U/l)	3 (75%)
> 1xULN	1 (25%)
Viral load (IU/mL) (median)	137,605
Fibrosis stage	
F0-F1	3 (75%)
F2-F3	1 (25%)
Management advice	
No therapy	1 (25%)
Antiviral therapy	3 (75%)

*WE* Western Europe, *EE* Eastern Europe, *EA* Euro Asia, *ALT* Alanine-aminotransferase, *ULN* Upper limit of normal

Only 4/20 (20%) of invited HCV patients responded to the invitation and were evaluated at our clinic and 3/4 (75%) started antiviral therapy.

## Discussion

The NHHRP was initiated to retrieve chronic HBV and HCV patients without follow-up in primary or specialist care. We aimed to identify these patients and invite them for re-evaluation at our clinic.

The number of patients eligible for retrieval for both HBV and HCV was considerably lower than the total number of patients lost to follow-up. The major reason for this drawback was that updated contact details were lacking. About one-third of those identified to lost to follow-up moved to another region and may have received adequate follow-up and care elsewhere. Due to national privacy regulations we were not allowed to search for updated data outside our own medical records, such as municipal databases. We expect this privacy regulation to be a major limitation in future retrieval projects. However, in Iceland, chronic HCV was defined as a public health threat and therefore a nationwide elimination programme, treatment as prevention for hepatitis C in Iceland (TRAP HEP C), was launched. Unlike our approach, in the context of this TRAP HEP C programme it was allowed to check updated contact details in the municipal database and to contact the patient directly [11].

Among the patients not eligible for retrieval we identified two important groups. The first group consisted of asylum seekers whose tests were performed in asylum seeker centers upon arrival. At the time of our retrieval project current address and legal status were unknown and therefore we were not able to retrieve and re-evaluate this group. Our numbers suggest that this group could be a significant target group for retrieval and with better information and cooperation between asylum seeker centers and hepatitis treatment centers we could offer this group a chance of control or cure of their disease.

Second, prisoners tested positive during detention period are lost to follow-up after transferal to other detention centers or release to freedom without organizing follow-up in their place of residence. With close cooperation between prisons and hepatological centers this group is especially suitable for further evaluation and treatment of their chronic HBV or HCV. A large state-wide programme in Australia showed that screening and treatment of prisoners can be very successful if it is done in a structured approach [12]. However, reimbursement of diagnostics and therapy within the detention period differs per country and could be a challenge.

Only 44% of chronic HBV patients eligible for retrieval was referred for re-evaluation. A lack of awareness of both patients and primary health care physician is a possible explanation. Awareness about chronic HBV and HCV can be created through education or media campaign. Increased awareness will contribute to the effect of a retrieval programme and may enhance the willingness of target groups to participate in these programmes.

The major change of management in chronic HBV patients was strict surveillance of the patient and to a lesser extent indication for antiviral therapy. Evaluation resulted in a major change of management in 44% of the patients. The remaining 50% had an indication for 6-12 monthly check of ALT levels and viral load. Patients were evaluated using the 2012 Dutch guideline on chronic HBV infection as standard. In 2017 the European Association of Studies of the Liver (EASL) released the updated guideline on HBV infection. This guideline sets a stricter cut-off point for indications for treatment with all patients with HBeAg-positive or –negative chronic hepatitis B, a viral load of > 2.0 • 10^3^ in combination with ALT greater than the upper limit of normal and/or at least moderate liver necroinflammation or fibrosis should be treated.

Three HBV patients started antiviral therapy based on viral loads in combination with elevated ALT levels. An additional 5 patients had an indication for strict follow-up because of elevated ALT levels or fibrosis stage F2-F3 despite viral load < 2,0 • 10^4^. These patients now may have an indication for therapy if HBV DNA levels are > 2,0 • 10^3^ according to the updated EASL guideline [7].

Concerning HCV patients, the results of retrieval were disappointing. We observed that two-third of patients had treatment or adequate follow-up, but the remaining one-third was hard to retrieve due to above-mentioned reasons. Moreover, people who inject drugs (PWID) are an important target group as well. Because this group often has no permanent address, it is especially hard to reach them. However, treatment of PWID can be successfully in a multidisciplinary setting using strategies such as directly observed therapy and the involvement of nurse-practioners [13]. In close cooperation with addiction centers this group is particularly suitable for structured screening and therapy, for instance in conjunction with opioid substation therapy.

Despite difficulties with reaching above mentioned target groups, the effect of retrieval of chronic HCV patients will be significant. As of October 2015 direct antiviral agents for hepatitis C are approved for reimbursement of every basic health insurance in the Netherlands. Therefore, according to the Dutch guideline on hepatitis C, every patient with HCV infection has an indication for antiviral therapy. We started antiviral therapy in 3 of 4 patients, in one patient we did not start because of limited life expectancy.

A considerable larger number of HBV patients was lost to follow-up compared to HCV patients. The population of HBV patients predominantly consisted of migrants whereas the HCV population mainly consisted of PWID. It is possible that migrants have more difficulties with access to health care whilst PWID often have follow-up in addiction care and are easily referred to health care. Furthermore, in the past need for treatment and follow-up for chronic HBV infection was less strict compared to HCV patients and HBV patients therefore easily got lost to follow-up.

The most time-consuming element was the complicated construction to contact patients via their primary health care physician. Our region is a low-endemic region for HBV and HCV and most practices only have one or two HBV or HCV patients. Therefore, viral hepatitis is not a priority to most primary health care physicians. If it would be possible to approach the patients directly by using updated contact details, a retrieval project could be much more effective to perform. But we expect that, due to (inter) national privacy regulations, a direct approach will be hard to implement.

Our retrieval project is currently expanded to other regions in the Netherlands. The results of these projects will show us if large scale retrieval projects are worth the effort.

## Conclusions

We conclude that a retrieval project comparing datafiles of the microbiological laboratory and medical records is time-consuming and will only lead to a limited percentage of patients lost to follow-up eligible for retrieval. Nonetheless it will lead to a change of management in a significant percentage of evaluated patients. Therefore, structured retrieval programmes of chronic HBV and HCV are an important element in the accomplishment of the WHO goal to eliminate HBV and HCV as public health care threat.

## Abbreviations

ALT: Alanine-aminotransferase
EASL: European association of studies of the liver
HBeAg: Hepatitis B e antigen
HBsAg: Hepatitis B surface antigen
HBV: Hepatitis B virus
HCV: Hepatitis C virus
NHHRP: Northern Holland hepatitis retrieval project
PWID: People who inject drugs
WHO: World Health Organisation
